# CoIN: co-inducible nitrate expression system for secondary metabolites in *Aspergillus nidulans*

**DOI:** 10.1186/s40694-018-0049-2

**Published:** 2018-03-13

**Authors:** Philipp Wiemann, Alexandra A. Soukup, Jacob S. Folz, Pin-Mei Wang, Andreas Noack, Nancy P. Keller

**Affiliations:** 1 Department of Medical Microbiology and Immunology, University of Wisconsin, Madison, WI 53706 USA; 2 Department of Bacteriology, University of Wisconsin, Madison, WI 53706 USA; 3 Present Address: Hexagon Bio, Menlo Park, CA 94025 USA; 4 Present Address: Department of Cell and Regenerative Biology, University of Wisconsin, Madison, WI 53705 USA; 5 Present Address: Davis Genome Center – Metabolomics, University of California, 451 Health Science Drive, Davis, CA 95616 USA; 6 Present Address: Ocean College, Zhejiang University, Hangzhou, 310058 Zhejiang Province People’s Republic of China

**Keywords:** Yeast recombinational cloning, Secondary metabolism, Genetic engineering, Carotenes, *Aspergillus nidulans*, *Fusarium fujikuroi*, AflR, Sterigmatocystin, Nitrate, Biotechnology, Synthetic biology

## Abstract

**Background:**

Sequencing of fungal species has demonstrated the existence of thousands of putative secondary metabolite gene clusters, the majority of them harboring a unique set of genes thought to participate in production of distinct small molecules. Despite the ready identification of key enzymes and potential cluster genes by bioinformatics techniques in sequenced genomes, the expression and identification of fungal secondary metabolites in the native host is often hampered as the genes might not be expressed under laboratory conditions and the species might not be amenable to genetic manipulation. To overcome these restrictions, we developed an inducible expression system in the genetic model *Aspergillus nidulans*.

**Results:**

We genetically engineered a strain of *A. nidulans* devoid of producing eight of the most abundant endogenous secondary metabolites to express the sterigmatocystin Zn(II)_2_Cys_6_ transcription factor-encoding gene *aflR* and its cofactor *aflS* under control of the nitrate inducible *niiA*/*niaD* promoter. Furthermore, we identified a subset of promoters from the sterigmatocystin gene cluster that are under nitrate-inducible AflR/S control in our production strain in order to yield coordinated expression without the risks from reusing a single inducible promoter. As proof of concept, we used this system to produce β-carotene from the carotenoid gene cluster of *Fusarium fujikuroi*.

**Conclusion:**

Utilizing one-step yeast recombinational cloning, we developed an inducible expression system in the genetic model *A. nidulans* and show that it can be successfully used to produce commercially valuable metabolites.

**Electronic supplementary material:**

The online version of this article (10.1186/s40694-018-0049-2) contains supplementary material, which is available to authorized users.

## Background

Natural products or secondary metabolites (SMs) have been invaluable as platforms for developing front-line drugs. Between 1981 and 2010, 5% of the 1031 new chemical entities approved as drugs by the Food and Drug Administration (FDA) were natural products or derivatives, including 48.6% of cancer medications [36]. In addition, SMs are major sources of innovative therapeutic agents for both bacterial and fungal infectious diseases, lipid disorders, and immunomodulation [16]. Fungal SMs have proven to be a particularly important source of new leads with useful pharmaceutical activities. A literature survey of fungal metabolites, covering 1500 fungal SMs that were isolated and characterized between 1993 and 2001, showed that more than half of the molecules had antibacterial, antifungal or antitumor activity [41]. However, the full metabolic potential of the majority of existing fungal species has not been investigated. Major roadblocks in this endeavor are that some species are not cultivable under laboratory conditions and/or their SM gene clusters are silent. Previous strategies on activating fungal SMs have focused mainly on (1) activating endogenous gene clusters by over-expressing the pathway-specific transcription factor [13, 43, 54]), (2) manipulating global regulators [11, 28], and (3) expressing the entire gene cluster in a heterologous host [8]. Although successful in some cases, these strategies have significant disadvantages. As not all fungal species are easily amenable to genetic manipulation, strategies that focus on endogenous activation are impossible in these species. This prevents the option of over-expressing a cluster-specific transcription factor, which has been the most successful approach to activating cryptic clusters thus far (reviewed in [51]). In addition, not all SM clusters contain transcription factors and although some clusters have been activated by overexpressing every gene in the cluster [14, 53], this adds labor and time to the process and may be hard to achieve with clusters containing many genes.

Previous approaches expressing fungal gene clusters in heterologous hosts (mainly *Saccharomyces cerevisiae* or *Aspergillus* spp.) focused on amplification of the entire gene cluster including native promoters. Although these approaches lead to expression of the targeted gene clusters in some cases [55], the use of native promoters cannot guarantee controlled activation of the genes. Therefore, the identification and use of defined promoters presents an alternative means to activate clusters. Cloning of entire gene clusters can be achieved by PCR-based amplification of the desired DNA region and subsequent yeast recombination-based cloning [55]. Promoter exchanges using this technique rely on the identification of different promoter regions as the use of identical promoter sequences is impossible due to the homologous recombination among promoters [14].

Our goal was to identify a series of distinct promoters that could be activated in one step and, furthermore, activated under an inducible system as many SMs exhibit antifungal properties that could be toxic to the heterologous host [12, 46]. Thus, we designed a strain of genetic model organism *Aspergillus nidulans* that contains an inducible genetic construct which allows for expression of the positive acting transcriptional elements of the sterigmatocystin (ST) gene cluster, *aflR* and *aflS* (formerly *aflJ*, [20]). The ST gene cluster contains 25 distinct genes and it is known that the transcription factor AflR and its cofactor AflS are responsible for ST production [9]. We constructed a strain of *A. nidulans* with its endogenous ST cluster removed but with *aflR*/*aflS* placed back into the strain under the control of a nitrate inducible divergent promoter (*niiA*(p)/*niaD*(p)) [44, 45], thereby allowing controlled *aflR*/*aflS* expression based on culture conditions. We tested the expression of all 25 ST promoters by AflR/AflS in this strain and identified eight ST promoters specifically regulated by nitrate induction of *aflR/S*. To test the system for expression of a fungal secondary metabolite, we cloned the carotenoid gene cluster from *Fusarium fujikuroi* and placed it under control of these inducible ST promoters. We show that the derived *A. nidulans* transformants produce β-carotene in competitive levels to existing systems using our technology.

## Methods

### Fungal strains and culture conditions

**Fig. 1:**
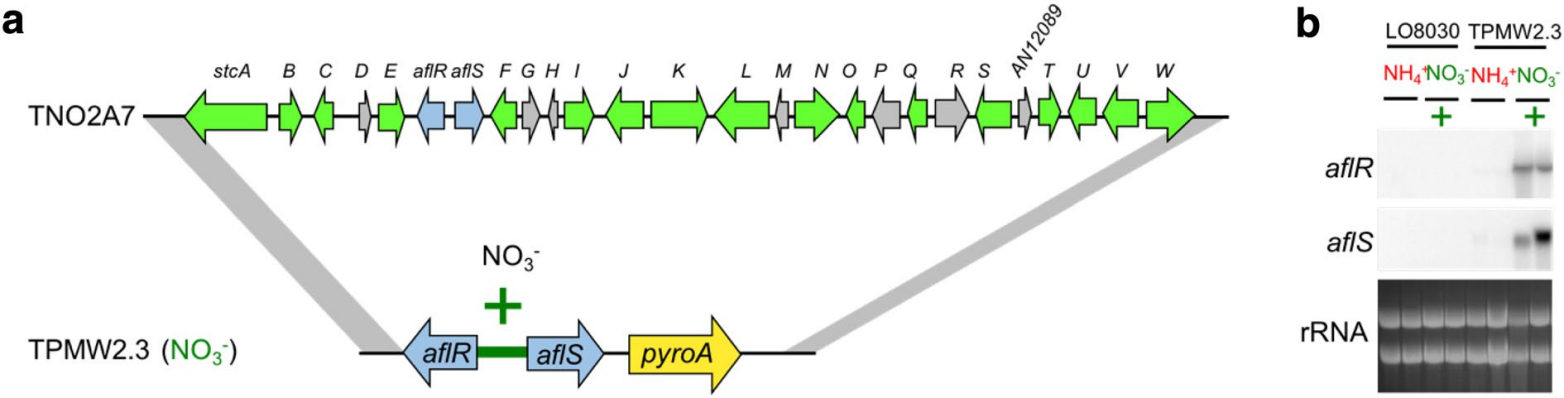
Nitrate-inducible *aflR/S* expression in *A. nidulans.* **a** Schematic overview of the *aflR*/*S* expression strain TPMW2.3 that harbors the nitrate-inducible *niaD*/*niiA* promoter-driven *aflR*/S genes at the native sterigmatocystin cluster locus. **b** Northern blot analysis of nitrate-dependent *aflR*/*S* expression in *A. nidulans* TPMW2.3. Strains were grown in 50 mL of GMM with 35 mM glutamine as nitrogen source supplemented with 5 mM uracil/uridine and riboflavin for 24 h at 250 rpm at 37 °C. The mycelia were washed and shifted into new media containing either NH_4_^+^ or NO_3_^−^ as nitrogen sources plus supplements and grown at 250 rpm and 37 °C for 1 h before RNA extraction. Strains were grown in duplicate, indicated genes were probed and rRNA was visualized as loading control

*Aspergillus nidulans* strains used in this study are listed in Additional file 1: Table S1. *Fusarium fujikuroi* IMI58289 [51] was used for *carRA*, *carB*, and *ggs1* amplification as a reference for carotenoid production. Strains were maintained as glycerol stocks and activated on solid glucose minimal medium (GMM) at 37 °C with appropriate supplements [48]. For experiments in Fig. 1, nitrate was replaced with equimolar ammonium tartrate and supplemented with 5 mM uracil and uridine, respectively. For solidified media, Noble Agar (Difco™, BD, USA) was added at 16 g/L. For *pyrG* auxotrophs, the growth medium was supplemented with 5 mM uridine and uracil. For *riboB* auxotrophs, the growth medium was supplemented with 5 mM riboflavin. For *pyroA* auxotrophs, the growth medium was supplemented with 5 mM pyridoxine. Conidia were harvested in 0.01% Tween 80 and enumerated using a hemocytometer. For RNA analysis, indicated strains were inoculated into 50 mL of liquid GMM with 35 mM glutamine as nitrogen source at 5 × 10^6^ conidia/mL in duplicate and grown at 37 °C and 250 rpm for 24 h in ambient light conditions. The cultures were shifted into new GMM media either containing 70 mM nitrate or 35 mM glutamine as nitrogen source for 1 h. The mycelium was harvested and lyophilized before RNA extraction. For carotenoid production 5 × 10^6^ conidia of indicated strains were inoculated on 20 mL liquid stationary GMM with either containing 70 mM nitrate or 35 mM glutamine as nitrogen source for 3 days at 37 °C (*A. nidulans*) or 29 °C (*F. fujikuroi*) in the dark. To distinguish between carotenoid production in the mycelia and spores, the strains were grown on liquid stationary GMM media (described above) for 3 days at 37 °C in the light to induce sporulation. Mycelia and spores were resuspended in 0.01% (v/v) Tween80, vortexed to separate spores from mycelia. Spores were separated from mycelia by filtration.

### Yeast recombinational cloning

Yeast strain BJ5464 (*MATalpha*, *ura3*-*52*, *trp1*, *leu2*-*∆1*, *his3*-*∆200*, *pep4::HIS3*, *prb1*-*∆1.6R*, *can1*, *GAL*) was inoculated into 25–50 mL of appropriate media (2× YPDA) and incubated at 30 °C at 200 rpm overnight. The concentration of overnight culture was determined using OD 600 with a 1 × 10^7^ cells/mL set to an OD reading of 1.0. Then, 1.25 × 10^9^ cells were centrifuged at 3000×*g* for 5 min. Fresh media was added to the pelleted cells and added to a baffled flask containing 250 mL of 2× YPAD to a final concentration of 5 × 10^6^ cells/mL and incubated at 30 °C at 200 rpm until the cell titer reached 2 × 10^7^ cells/mL. Cells were harvested by centrifugation at 3000×*g* for 5 min. Supernatant was removed and the cells were washed with double distilled H_2_O (ddH_2_O). The cells were transferred to one 50 mL falcon tube and washed an additional time with ddH_2_O before they were centrifuged at 3000×*g* for 5 min. Cells were resuspended in 5% glycerol and 10% DMSO to a final concentration of 2 × 10^9^ cells/mL aliquoted in 100 µL. These cells can be frozen at − 80 °C for several weeks. Before transformation, cells were pelleted and the supernatant removed. For transformation 250 ng of the digested backbone vector and 500 ng of each DNA PCR product (see below) were added and adjusted to a final volume of 14 µL with ddH_2_O. The DNA mixture was added to the yeast along with 260 µL of a 50% (w/v) polyethyleneglycol 3600, 36 µL 1 M lithium acetate and 50 µL of denatured sheared salmon sperm DNA (2 mg/mL). The mixture was vortexed and incubated at 42 °C for 45 min. Cells were centrifuged at 13,000×*g* for 30 s and the supernatant was removed. The cells were carefully resuspended in 1 mL ddH_2_O and 200–500 µL were spread on solidified synthetic drop out media containing all necessary supplements without uracil for selection. Plates were incubated at 30 °C for 3–5 days.

### Plasmid isolation from yeast

All colonies from a transformation plate were scraped and incubated overnight in liquid synthetic yeast drop out solution containing the appropriate supplements without uracil at 200 rpm at 30 °C. One mL was pelleted and the supernatant removed. 200 µL of STC buffer (50 mM Tris–HCl pH 7.5, 1.2 M sorbitol, 50 mM CaCl_2_) including 3 µL Zymolase was added and incubated at 37 °C for 1 h. To the mixture, 200 µL of 1% (w/v) sodium dodecyl sulfate (SDS) in 200 mM NaOH were added and inverted. The solution was neutralized by adding 240 µL of 3 M potassium acetate, pH 5.5 and inverted. The mixture was centrifuged and the supernatant mixed with 600 µL isopropanol, inverted and centrifuged at maximum speed for 10 min. The supernatant was removed and the pellet was washed with 70% (v/v) ethanol. The pellet was air dried and resuspended in 30 µL ddH_2_O.

### Transformation of *Escherichia coli* and plasmid conformation

Following standard techniques [23], 10 µL of the yeast plasmid extract were transformed into *E. coli* and positive colonies were selected on media containing ampicillin. Plasmids from colonies were isolated using standard procedures [23]. Plasmids were restriction digested with appropriate enzymes to confirm correct insertion. Additional confirmation was achieved using PCR amplification of fused DNA products. To ensure correct DNA sequences for expression plasmids, Sanger sequencing was performed. The correct plasmids were then grown in a 50 mL culture and plasmids were isolated using the Quantum Prep^®^ Plasmid Midiprep Kit (Biorad) according to the manufacturers’ instructions. Before fungal transformation, the plasmids were linearized using *Asc*I.

### Plasmid construction and fungal transformation

Expression fragments were created by yeast recombinational cloning as described above. All primers used are listed in Additional file 1: Table S2 and all plasmids are listed in Additional file 1: Table S3. For assembling the nitrate inducible *aflR*/*S* construct, six fragments total were amplified and eventually cloned into the *Asc*I digested plasmid backbone of pYHC-yA-riboB [57]. The 3′ flanks of *stcA* and *stcW* were amplified from *A. nidulans* LO8030 DNA using primer pairs *stcA*3′-F/-R and *stcW*3′-F/-R with the -R primers containing 5′ overlaps to the respective site of *Asc*I digested plasmid backbone and the -F primers having overlaps to the *aflS* terminator and *pyroA* cassette, respectively. The bidirectional *niaD*/*niiA* promoter region was amplified from *A. nidulans* LO8030 [15, 38] with overlaps to the open reading frames of *aflR* and *aflS* using primer pairs nitrate-F/-R. The open reading frame of *aflR* including 500 bp of terminator was amplified from *A. nidulans* FGSC 4A DNA using primer pair *aflR*-F/-R where the -R primer had a 5′ overhang to the terminator region of the *A. fumigatus pyroA* gene. The open reading frame of *aflS* was amplified from *A. nidulans* FGSC 4A DNA using primer pairs *aflS*-F/-R with the -R primer having a 5′ overlap to the -F primer used to amplify the *stcW* flank. The *pyroA* cassette was retrieved through *Pst*I restriction digest of pJMP61 [7]. After yeast recombinational cloning, the plasmid pPMW1 was created. For sterigmatocystin promoter studies the entire bidirectional promoter region between two open reading frames was cloned or, in the case of monodirectional promoters 500 bp upstream of the open reading frame was amplified using primer pairs *stc* “gene name”-pF/*stc* “gene name”-pR including 5′ overlaps to the *wA* 5′ flank and the open reading frame of *pyrG* gene from *A. fumigatus* CEA10. Plasmid pYHC-wA-pyrG [55] was linearized using *Nhe*I. After yeast recombineering, plasmid pAN “stcGene” were yielded. For constructing the carotenoid expression plasmid pJSF1, the bidirectional promoter region between *stcA* and *stcB* was amplified using primer pair *stcAB*-cF/-cR with overlaps to the carotenoid cluster genes *carRA* and *carB* from *F. fujikuroi* IMI58289. The open reading frame of *carRA* including 500 bp terminator region was amplified from *F. fujikuroi* IMI58289 DNA using primer pairs *carRA*-F/-R with the -R primer including an overlap to the *wA* 3′ flank of plasmid pYHC-wA-pyrG. The open reading frame of *carB* including 500 bp terminator region was amplified from *F. fujikuroi* IMI58289 DNA using primer pairs *carB*-cF/-cR with the -cR primer including an overlap to the *wA* 5′ flank of plasmid pYHC-wA-pyrG. All fragments were assembled using yeast recombinational cloning into *Eco*RI/*Xho*I linearized pYHC-wA-pyrG resulting in pJSF1. pJSF2 was assembled in a similar process using EcoRI/XhoI linearized pYHC-yA-riboB and PCR amplicons of the *stcM* promoter (amplified with *stcM*-*cF/*-*cR*) and *ggs1* (amplified with *ggs1*-*cF/*-*cR*).

Transformation of *A. nidulans* was performed as previously described [40]. For selection of nitrate inducible *aflR*/*S* strains, *A. nidulans* LO8030 was used as the recipient strain. pPMW1 was linearized using *Asc*I, transformed into LO8030, and transformants were selected on media where pyridoxine was omitted and uracil/uridine and riboflavin were supplemented yielding strain TPMW2.3. For selection of *stc* promoter test strains, TPMW2.3 was used as the recipient strain. pAN “stcGene” plasmids were linearized using *Sbf*I and transformants were selected on media were riboflavin was omitted and uracil/uridine was supplemented yielding strains TANx and TAASx (see Additional file 1: Table S1). To create a riboflavin prototrophic strain, TPMW2.3 was transformed with *Sbf*I linearized pYHC-yA-riboB and selected on media where riboflavin was omitted and uracil/uridine was supplemented yielding strain TPMW7.2. For selection of *car* expression strains, TPMW7.2 was used as the recipient strain and *Sbf*I linearized pTJSF1 was transformed into TPMW7.2 and transformants selected on media omitting uracil/uridine yielding strain TJSF1.1. An auxotrophic control strain was generated by using TPMW7.2 as recipient strain and *Sbf*I linearized pYHC-wA-pyrG was transformed and selected on media without supplements yielding strain TPMW8.2. For DNA isolation, all fungal strains were grown for 24 h at 37 °C (*Aspergillus*) or 29 °C (*Fusarium*) in steady state liquid GMM, supplemented appropriately as described by Shimizu and Keller [48]. Single integration was confirmed by Southern analysis as described by [23] using P^32^-labelled probes created by amplification of the indicated DNA fragment in Additional file 2: Figures S1–S4.

### Carotenoid analysis

Carotenoids were extracted and analyzed as previously described [18, 19]. Briefly, carotenoids were extracted with acetone from freeze dried mycelia and were separated by thin layer chromatography developed in light petroleum/diethyl ether/acetone (4:1:1; v/v/v). The bands were scraped out and dissolved in acetone. High-performance liquid chromatography (HPLC) was used to analyze the β-carotene content by comparison to an authentic standard. HPLC separation was performed on using a ZORBAX Eclipse XDB-C18 column (Agilent, 4.6 mm by 150 mm with a 5 μm particle size) by using a binary gradient of methanol/t-butylmethyl ether (1:1) (v/v) as solvent A and methanol/t-butylmethyl ether/water (5:1:1) (v/v/v) as solvent B using a Flexar Binary Liquid Chromatography (LC) Pump (PerkinElmer) coupled to a Flexar LC Autosampler (Perkin Elmer) and a Flexar PDA Plus Detector (PerkinElmer). The binary gradient started with a linear step from 0 A to 57% A in 45 min and an additional linear gradient to 100% A in 0.5 min and hold for 25 min at a flow rate of 2 mL/min. Identification and relative quantification of secondary metabolites was performed using Chromera Manager (PerkinElmer) by comparison to an authentic standard (Sigma Aldrich).

## Results

The sterigmatocystin (ST) gene cluster of *A. nidulans* is known to harbor 25 genes involved in biosynthesis of sterigmatocystin [9]. While environmental regulation of the ST gene cluster is complex and not well understood, it was the first cluster that identified a gene product encoded within the cluster itself to function as a cluster-specific Zn(II)_2_Cys_6_ transcription factor, called AflR [21]. The gene encoding AflR shares a bidirectional promoter with *aflS* encoding a transcriptional cofactor of AflR [20]. We replaced the native promoter of *aflR*/*S* with the well characterized *niaD*/*niiA* promoter which is induced by the presence of nitrate in the absence of other nitrogen sources [10]. We confirmed nitrate-dependent expression of *aflR*/*S* by northern blot analysis (Fig. 1b).

**Fig. 2:**
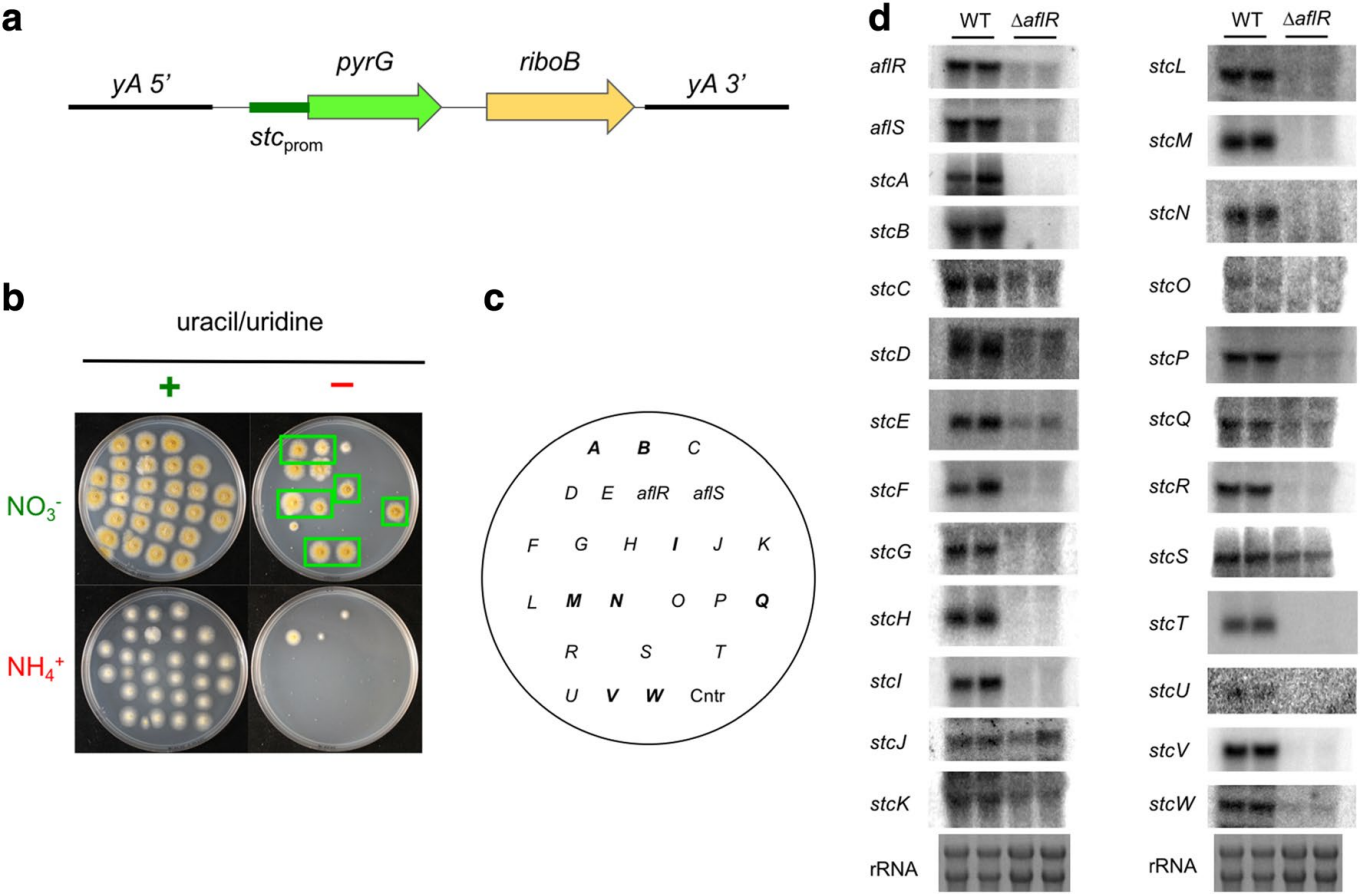
Nitrate- and *stc* promoter-dependent *pyrG* expression. **a** Schematic overview of *A. niduans stc* promoter driven *pyrG* test strains integrated at the *yA* locus. **b** *A. nidulans* strains were grown for 72 h at 37 °C on solidified GMM plates containing NH_4_^+^ or NO_3_^−^ as nitrogen and half of the plates were supplemented with 5 mM uracil/uridine as indicated. Green boxes indicate promoters with desirable traits of strict nitrate inducibility. **c** Schematic overview of spotting pattern of strains containing the indicated *stc* promoter driving *pyrG* on the plates depicted in panel (**b**). Bold letters indicate *stc* promoters with desired traits of nitrate inducibility. Cntr is the control strain TPMW7.2. **d** Northern blot analysis of all sterigmatocystin cluster genes from the *A. nidulans* WT and a ∆*aflR* knock-out mutant. Strains were grown in duplicate under sterigmatocystin production conditions (in 50 mL of GMM at 37° for 48 h at 250 rpm) before RNA extraction. Probes for indicated genes were hybridized and rRNA was visualized as loading control

Next, we set out to test which of the 25 *stc* gene promoters would be nitrate-inducible in our production strain (TPMW2.3). Since TPMW2.3 is a uracil/uridine and riboflavin auxotroph, we designed plasmids that contain each of the 25 *stc* gene promoters, respectively, driving expression of the *A. fumigatus pyrG* gene along with a *riboB* selectable marker flanked by bordering regions of the *yA* locus (Fig. 2a; Additional file 1: Table S3; Additional file 2: Fig. S2). Using a minimalized promoter selection strategy, we chose promoter regions as follows: For unidirectional *stc* genes, the promoter region was amplified from the first base after the stop codon of the first gene to the start codon of the second gene, but not exceeding 1 kb. In cases of bidirectional promoters, the entire region between the two start codons was chosen, not exceeding 1 kb. We selected 25 strains for each *stc* promoter for riboflavin prototrophy, exhibiting yellow spore color, and a control strain that did not include a *stc* promoter. To test for the ability of AflR/S to induce Af*pyrG* expression driven by each of the *stc* promoters, we grew them on media containing either nitrate (induces *aflR*/*S* expression; Fig. 1b) or ammonium, and supplemented with or without uracil/uridine (Fig. 2b, c). The growth assay showed that eight of the tested promoters (*stcA*, *stcB*, *stcI*, *stcM*, *stcN*, *stcQ*, *stcV*, and *stcW*) exhibited the desired ability to grow on plates without uracil/uridine supplementation on nitrate containing media only (Fig. 2b, c), demonstrating specific expression under induction conditions. Three of the promoters tested exhibited leaky expression (*stcC*, *stcD*, and *stcE*) as we observed colony growth of strains on media containing ammonium (Fig. 2b, c) where *aflR*/*S* should not be induced (Fig. 1b). In order to confirm control of the identified promoters by AflR, we investigated expression of all *stc* cluster genes in an *A. nidulans* WT and an isogenic ∆*aflR* knock-out strain [56] under sterigmatocystin production conditions. We found that in addition to the eight promoters identified in our plate assay, most of the remaining cluster genes were also expressed in an AflR-dependent manner (Fig. 2d). We speculate that either the length of the chosen promoters, the difference in culture conditions, or the insufficient expression level could be responsible for the observed discrepancies of *stc* activation of the *AfpyrG* reporter gene.

**Fig. 3:**
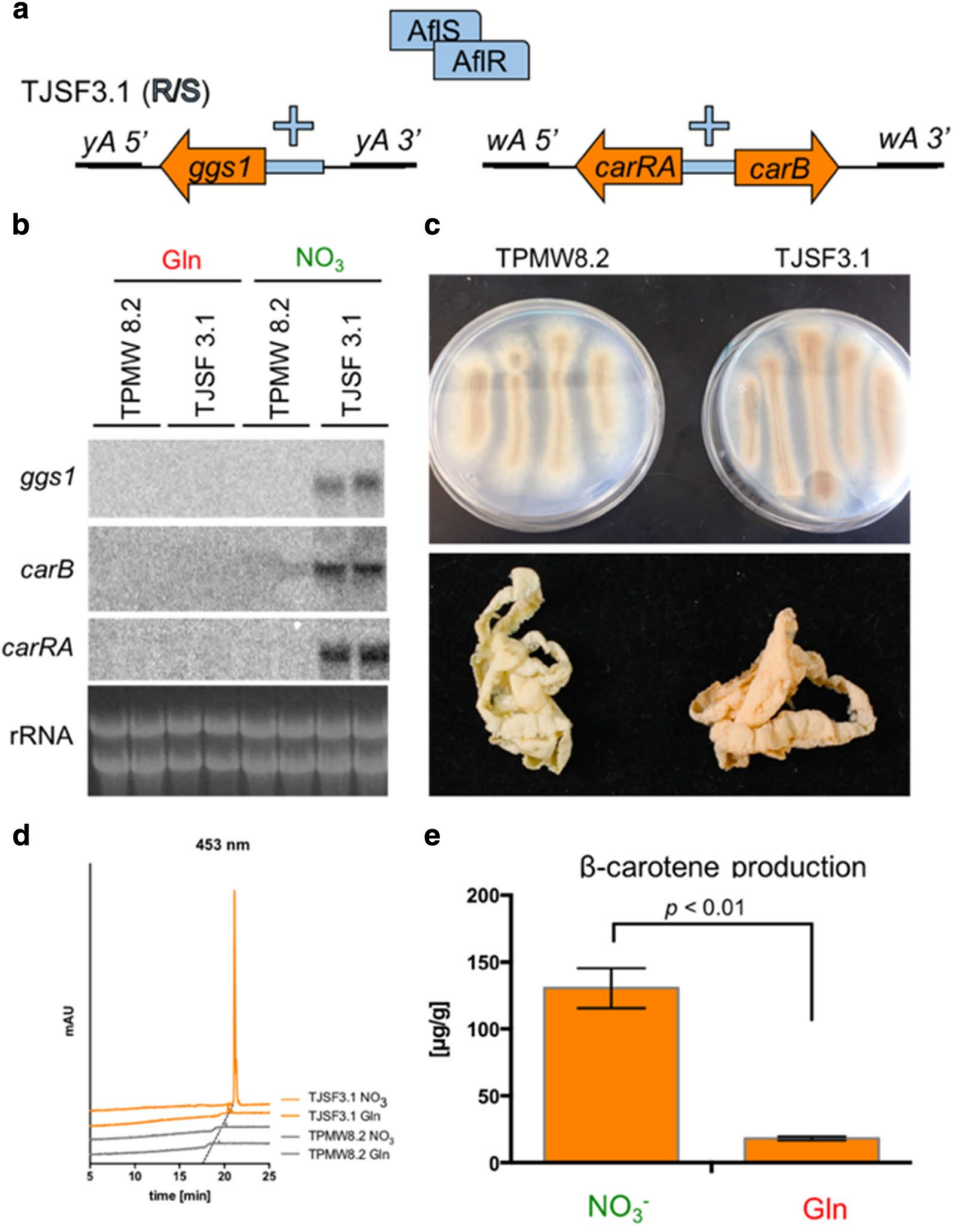
Nitrate-dependent β-carotene production in *A. nidulans.* **a** Schematic overview of *A. nidulans* carotenoid production strain TJSF3.1 that harbors the *stcM*-driven *F. fujikuroi ggs1* gene at the *yA* locus and the *stcA*/*B*-driven *F. fujikuroi carRA*/*B* genes at the *wA* locus. **b** Northern blot expression analysis of indicated genes in the indicated strains. The strains were grown in duplicate for 24 h in 50 mL of GMM with 35 mM glutamine (Gln) as nitrogen source for 24 h at 250 rpm at 37 °C. The mycelia were washed and shifted into new media containing either Gln or NO_3_^−^ as nitrogen sources and grown at 250 rpm and 37 °C for 1 h before RNA extraction. RNA was visualized as loading control. **c** Growth on solid medium demonstrating carotene expression from strain TJSF3.1 and control strain TPMW8.2 grown for 72 h at 37 °C on solidified GMM media containing nitrate as nitrogen source. Bottom picture shows mycelia of the same strains collected from liquid stationary GMM media containing nitrate as nitrogen source grown for 72 h at 37 °C. **d** HPLC chromatograms at 453 nm of β-carotene extracted from indicate strains grown as described in panel (**c**). **e** Quantification of β-carotene produced by TJSF3.1 grown on either NO_3_^−^ or glutamine (Gln)

To test the functionality of our expression system genes responsible for carotenoid production from *Fusarium fujikuroi* [2–4] were expressed in our *A. nidulans* nitrate-inducible *aflR*/*S* strain TPMW2.3. In *F. fujikuroi*, the geranylgeranyl diphosphate (GGDP) synthase gene *ggs1* is responsible for production of GGDP [34], which is a substrate for CarRA and CarB, encoded by two of the clustered carotenoid biosynthetic genes needed for β-carotene production [31]. We inserted the *ggs1* gene driven by the *stcM* promoter and 0.5 kb of the native terminator, a riboflavin selectable marker flanked by the *yA* border regions (Additional file 1: Table S3; Additional file 2: Fig. S3), and the two carotenoid cluster genes *carRA* and *carB* including 0.5 kb of the native terminator regions, responsible for β-carotene production [31] under control of the bidirectional *stcA*/*B* promoter flanked by the *wA* border regions (Additional file 1: Table S3; Additional file 2: Fig. S3). Both plasmids were linearized and transformed into TPMW2.3 consecutively, yielding strain TJSF3.1 (Fig. 3a; Additional file 1: Table S1; Additional file 2: Fig. S4). Nitrate-inducible expression of *ggs1*, *carRA*, and *carB* was confirmed by northern blot analysis compared to a prototroph control strain that produced white spores (TPMW8.2) (Fig. 3b). When the two strains were grown on nitrate containing media, TJSF3.1 exhibited a characteristic orange color that was absent in the control (Fig. 3c). Characterization of carotene production by HPLC showed that the strain TJSF3.1 produced 125 µg β-carotene per gram mycelial dry weight in our experimental setting (Fig. 3d, e). The production of β-carotene was significantly higher on nitrate induction media than on non-induction media containing glutamine (Fig. 3d, e). The control strain TPMW8.2 did not show any carotene production (Fig. 3d).

**Fig. 4:**
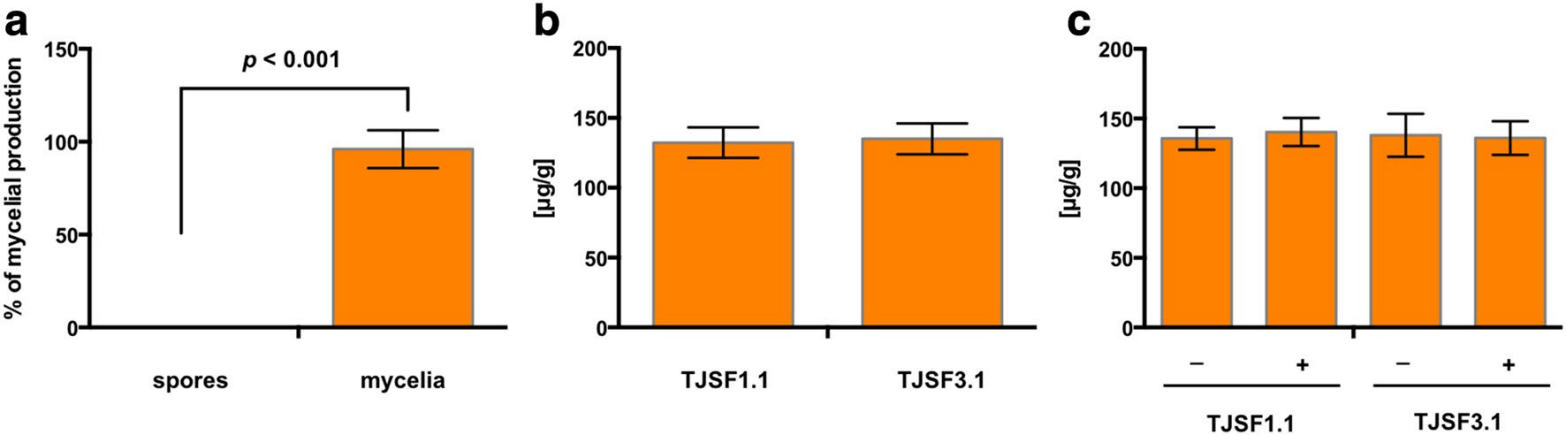
Tissue- and media-specific β-carotene production. **a** Comparison of β-carotene production in spores and mycelia of TJSF3.1 in triplicates. The strains were grown under nitrate inducing conditions and carotenoid production was normalized to the amount produced in mycelia. **b** Comparison of β-carotenoid production between TJSF1.1 (*carRA* and *carB*) and TJSF3.1 (*ggs1*, *carRA* and *carB*). Strains were grown under nitrate inducible conditions and β-carotene was quantified based on normalized dry weight in triplicates. No significant difference be could be detected. **c** Comparison of β-carotene production between TJSf1.1 (*carRA* and *carB*) and TJSF3.1 (*ggs1*, *carRA* and *carB*) grown on nitrate inducible conditions either supplemented with (+) or without (−) 10 mM mevalonate in triplicates. No significant difference be could be detected

As carotenoid production in *N. crassa* and *Fusarium* spp. occurs in both mycelia and spores [5], we asked whether a similar distribution would occur in our production strain. Therefore, mycelia and spores were assessed individually for β-carotene content. Carotenoids were only produced in the mycelia and not in the spores (Fig. 4a). Since the GGDP produce by Ggs1 is also utilized for ergosterol production in *F. fujikuroi* [34] we set out to investigate if the homolog of *ggs1* in *A. nidulans* (AN0654, 54% identity, *e*-value: 4.0^−118^) would be sufficient for β-carotene production. A strain was constructed that only expressed *carRA* and *carB* called TJSF1.1 (Additional file 2: Fig. S4). When carotenoid production between TJSF1.1 (*carRA* and *carB*) and TJSF3.1 (*ggs1*, *carRA*, and *carB*) was compared no significant difference under inducing conditions was observed (Fig. 4b), suggesting that AN0654 is sufficient to provide the maximum amount of GGDP that can be funneled into carotenoid production. As it is known that one of the bottlenecks during carotenogenesis is the production of mevalonate (a GGDP precursor) by the 3-hydroxyl-3-methyl-glutaryl-conenzyme A reductase (HMG CoA reductase) [1], we grew the two production strains on nitrate media supplemented with mevalonate before carotenoid quantification. However, we did not find any difference in production levels between the strains grown with or without mevalonate (Fig. 4c).

## Discussion

Many efforts have been made to increase expression of fungal natural products [6, 35]. Apart from increasing production in the native host, a major focus has been on developing heterologous expression systems. Heterologous systems have the advantage that they can be carried out in a safe host system without toxic byproducts, that is easily amenable to genetic manipulation and preferably inducible [50]. Traditional approaches are laborious as they are mainly based on over-expressing each natural product cluster individually or sequentially, thereby relying on multiple selection markers that limit the number of genes expressed and subsequently reduce the chemical complexity of the natural product produced [55]. There have been successful reports on marker recycling to overcome this issue [14, 38], but these approaches involve multiple time-consuming transformation steps. Additionally, construction of the expression plasmids or cassettes has been achieved by labor intensive restriction enzyme- or fusion PCR-mediated methods [22, 25]. The ease of yeast recombinational cloning [39] has been exploited for a wide range of molecular methods, including gene knock-out libraries [17] and expression systems [47] in filamentous fungi as well as yeast itself [8]. Technically, yeast recombinational cloning allows for the assembly of multiple PCR fragments up to a vector size of several ten thousand kilo bases [39]. One of the major hurdles to overcome during yeast recombineering, is undesired recombination among multiple identical DNA regions. Recently, a study in *A. terreus* demonstrated the requirement of an AflR-like transcription factor, TerR, for expression of all twelve terrein cluster genes [24] similar to our expression data for AflR-dependency of all 25 *stc* genes. The system was subsequently used to control one of the terrein promoters in a heterologous expression system in *A. niger* to demonstrate activation of *orsA* from *A. nidulans* [24]. Here, we have demonstrated the specific induction of eight of the 25 *stc* promoters to control the expression of a reporter gene (*pyrG*). Subsequently, we have utilized three of the eight promoters to successfully express three *Fusarium* spp. derived genes responsible for carotenoid production in *A. nidulans* and confirm functionality of their gene products by detection of β-carotene.

As a precursor of vitamin A, β-carotene has long been in the focus of biotechnology. The most prominent example of heterologous gene expression leading to the production of β-carotene is the development of Golden Rice by Monsanto [37]. However, β-carotenoid production was also achieved in baker’s yeast and bacteria. The amounts of carotenoids produced by the production strain constructed in this study are equivalent to the first production strain engineered in yeast [52]. Recent advances in manipulating metabolic pathways and genetic elements have increased the production in baker’s yeast [8, 30], and similar approaches could be undertaken to increase production in *A. nidulans*.

Notably, production of β-carotene is restricted to the mycelia in the *A. nidulans* production strains investigated in this study, whereas in other fungal species like *Neurospora crassa* and *F. fujikuroi*, carotenes are predominantly produced in asexual spores [26, 31]. One explanation might be the developmentally controlled expression pattern of the *niaD* promoter, as it was shown to be only transiently expressed at early stages of conidiation, but not at later time points [33]. Sterigmatocystin and aflatoxin in *Aspergillus* species are predominantly found in the mycelial fraction and a complex fusion network of vesicles containing different precursors and biosynthetic enzymes that ensure correct cellular localization of these secondary metabolites is being unveiled by several studies [32, 42, 49]. Additionally, pioneering work in *A. fumigatus* has demonstrated that certain natural products are predominantly produced in the asexual spores (conidia) [27, 29]. These findings suggest that conidial directed cellular pathways in the native host (*Fusarium*) may differ significantly from *Aspergillus* as location of β-carotene is not the same in the native and heterologous host. Determining which factors control the direction of fungal natural products to certain developmental structures in different fungal species will be a fascinating future task.

## Conclusions

This study presents a new heterologous expression system for fungal natural products in the genetic model organism *A. nidulans*. The system described here makes use of the ability to co-express, minimally, eight promoters by a fungal-specific Zn(II)_2_Cys_6_ transcription factor, AflR, and its cofactor AflS. By replacing the intrinsic bidirectional *aflR/S* promoter with a nitrate bidirectional inducible promoter, all eight identified genes can be simultaneously activated and repressed. As all eight identified promoters differ in their DNA sequence, the system has the potential to utilize one-step yeast recombinational cloning for assembly of entire secondary metabolite gene clusters. Here, we demonstrate the production of β-carotene by heterologous expression of three genes from *F. fujikuroi*. The inducibility of the system also is useful for production of toxic metabolites at a stage when the host strain has accumulated a significant biomass.

## Additional files

**Additional file 1.** Additional tables.

**Additional file 2.** Additional figures.

## Abbreviations

SM: secondary metabolite
FDA: Food and Drug Administration
ST: sterigmatocystin
