# Supplementary material for: CoIN: co-inducible nitrate expression system for secondary metabolites in Aspergillus nidulans
Source: Fungal Biol Biotechnol. 2018 Mar 13;5:6. doi: 10.1186/s40694-018-0049-2 (PMC5851313; doi:10.1186/s40694-018-0049-2)
Supplement: Supplementary file 1 — Additional file 1. Additional tables. [file 40694_2018_49_MOESM1_ESM.docx]

Supplementary Tables

**Table S1. Strains used in this study**

| **Strain ID** | **Genotype** | **Reference** | **Purpose** |
| --- | --- | --- | --- |
| TNO2A7 | *nkuA*Δ::*argB* *pyrG89 pyroA4 riboB*, *veA1* | (Nayak et al., 2006) | *A. nidulans* ST cluster control strain |
| LO8030 | *pyrG89*, *pyroA4*, *riboB2*, Δ*nkuA*::*argB*; *veA1*; Δ*ST*(AN7808-7825)::*pyrG*; Δ*emericellamide* (AN2445-2549)::*pyrG*; Δ*asperfuranone* (AN1039-1029)::*pyrG*; Δ*monodictyphenone* (AN10023-10021)::*pyrG*; Δ*terrequinone* (AN8512-8520)::*pyrG*; Δ*austinol1* (AN8379-8384)::*pyrG*; Δ*F9775A/B* (AN7906-7915)::*pyrG*; Δ*asperthecin* (AN6000-6002)::*pyrG*; Δ*austinol2* (AN9246-9259)::*pyrG*; Δ*pyrG* | (Chiang et al., 2016; Oakley et al., 2017) | expression surrogate |
| TPMW2.3* | *pyrG89*, *pyroA4*, *riboB2*, Δ*nkuA*::*argB*; *veA1*; ΔST::niiA/niaD(p)::alfR/S::pyroA | this study | nitrate inducible *aflR*/*S* strain |
| TAN1.1* | *pyrG89*, *pyroA4*, *riboB2*, Δ*nkuA*::*argB*; *veA1*; Δ*ST*::*niiA/niaD*(p)::*alfR/S*::*pyroA*; Δ*yA*::*stcA*(p)::*pyrG*::*riboB* | this study | *stcA* test strain |
| TAN2.1* | *pyrG89*, *pyroA4*, *riboB2*, Δ*nkuA*::*argB*; *veA1*; Δ*ST*::*niiA*/*niaD*(p)::*alfR*/*S*::*pyroA*; Δ*yA*::*stcB*(p)::*pyrG*::*riboB* | this study | *stcB* test strain |
| TAN3.1* | *pyrG89*, *pyroA4*, *riboB2*, Δ*nkuA*::*argB*; *veA1*; Δ*ST*::*niiA*/*niaD*(p)::*alfR*/*S*::*pyroA*; Δ*yA*::*stcC*(p)::*pyrG*::*riboB* | this study | *stcC* test strain |
| TAN4.1* | *pyrG89*, *pyroA4*, *riboB2*, Δ*nkuA*::*argB*; *veA1*; Δ*ST*::*niiA*/*niaD*(p)::*alfR*/*S*::*pyroA*; Δ*yA*::*stcD*(p)::*pyrG*::*riboB* | this study | *stcD* test strain |
| TAAS420.1* | *pyrG89*, *pyroA4*, *riboB2*, Δ*nkuA*::*argB*; *veA1*; Δ*ST*::*niiA*/*niaD*(p)::*alfR*/*S*::*pyroA*; Δ*yA*::*stcE*(p)::*pyrG*::*riboB* | this study | *stcE* test strain |
| TAN5.1* | *pyrG89*, *pyroA4*, *riboB2*, Δ*nkuA*::*argB*; *veA1*; Δ*ST*::*niiA*/*niaD*(p)::*alfR*/*S*::*pyroA*; Δ*yA*::*aflR*(p)::*pyrG*::*riboB* | this study | *aflR* test strain |
| TAN6.1* | *pyrG89*, *pyroA4*, *riboB2*, Δ*nkuA*::*argB*; *veA1*; Δ*ST*::*niiA*/*niaD*(p)::*alfR*/*S*::*pyroA*; Δ*yA*::*aflS*(p)::*pyrG*::*riboB* | this study | *sflS* test strain |
| TAAS421.1* | *pyrG89*, *pyroA4*, *riboB2*, Δ*nkuA*::*argB*; *veA1*; Δ*ST*::*niiA*/*niaD*(p)::*alfR*/S::*pyroA*; Δ*yA*::*stcF*(p)::*pyrG*::*riboB* | this study | *stcF* test strain |
| TAN7.1* | *pyrG89*, *pyroA4*, *riboB2*, Δ*nkuA*::*argB*; *veA1*; Δ*ST*::*niiA*/*niaD*(p)::*alfR*/*S*::*pyroA*; Δ*yA*::*stcG*(p)::*pyrG*::*riboB* | this study | *stcG* test strain |
| TAAS422.1* | *pyrG89*, *pyroA4*, *riboB2*, Δ*nkuA*::*argB*; *veA1*; Δ*ST*::*niiA*/*niaD*(p)::*alfR*/*S*::*pyroA*; Δ*yA*::*stcH*(p)::*pyrG*::*riboB* | this study | *stcH* test strain |
| TAAS423.1* | *pyrG89*, *pyroA4*, *riboB2*, Δ*nkuA*::*argB*; *veA1*; Δ*ST*::*niiA*/*niaD*(p)::*alfR*/*S*::*pyroA*; Δ*yA*::*stcI*(p)::*pyrG*::*riboB* | this study | *stcI* test strain |
| TAN8.1* | *pyrG89*, *pyroA4*, *riboB2*, Δ*nkuA*::*argB*; *veA1*; Δ*ST*::*niiA*/*niaD*(p)::*alfR*/*S*::*pyroA*; Δ*yA*::*stcJ*(p)::*pyrG*::*riboB* | this study | *stcJ* test strain |
| TAN9.1* | *pyrG89*, *pyroA4*, *riboB2*, Δ*nkuA*::*argB*; *veA1*; Δ*ST*::*niiA*/*niaD*(p)::*alfR*/*S*::*pyroA*; Δ*yA*::*stcK*(p)::*pyrG*::*riboB* | this study | *stcK* test strain |
| TAN10.1* | *pyrG89*, *pyroA4*, *riboB2*, Δ*nkuA*::*argB*; *veA1*; Δ*ST*::*niiA*/*niaD*(p)::*alfR*/*S*::*pyroA*; Δ*yA*::*stcL*(p)::*pyrG*::*riboB* | this study | *stcL* test strain |
| TAN11.2* | *pyrG89*, *pyroA4*, *riboB2*, Δ*nkuA*::*argB*; *veA1*; Δ*ST*::*niiA*/*niaD*(p)::*alfR*/*S*::*pyroA*; Δ*yA*::*stcM*(p)::*pyrG*::*riboB* | this study | *stcM* test strain |
| TAN12.1* | *pyrG89*, *pyroA4*, *riboB2*, Δ*nkuA*::*argB*; *veA1*; Δ*ST*::*niiA*/*niaD*(p)::*alfR*/*S*::*pyroA*; Δ*yA*::*stcN*(p)::*pyrG*::*riboB* | this study | *stcN* test strain |
| TAAS424.1* | *pyrG89*, *pyroA4*, *riboB2*, Δ*nkuA*::*argB*; *veA1*; Δ*ST*::*niiA*/*niaD*(p)::*alfR*/*S*::*pyroA*; Δ*yA*::*stcO*(p)::*pyrG*::*riboB* | this study | *stcO* test strain |
| TAAS425.1* | *pyrG89*, *pyroA4*, *riboB2*, Δ*nkuA*::*argB*; *veA1*; Δ*ST*::*niiA*/*niaD*(p)::*alfR*/*S*::*pyroA*; Δ*yA*::*stcP*(p)::*pyrG*::*riboB* | this study | *stcP* test strain |
| TAN13.1* | *pyrG89*, *pyroA4*, *riboB2*, Δ*nkuA*::*argB*; *veA1*; Δ*ST*::*niiA*/*niaD*(p)::*alfR*/*S*::*pyroA*; Δ*yA*::*stcQ*(p)::*pyrG*::*riboB* | this study | *stcQ* test strain |
| TAN14.1* | *pyrG89*, *pyroA4*, *riboB2*, Δ*nkuA*::*argB*; *veA1*; Δ*ST*::*niiA*/*niaD*(p)::*alfR*/*S*::*pyroA*; Δ*yA*::*stcR*(p)::*pyrG*::*riboB* | this study | *stcR* test strain |
| TAN15.1* | *pyrG89*, *pyroA4*, *riboB2*, Δ*nkuA*::*argB*; *veA1*; Δ*ST*::*niiA*/*niaD*(p)::*alfR*/*S*::*pyroA*; Δ*yA*::*stcT*(p)::*pyrG*::*riboB* | this study | *stcS* test strain |
| TAN16.1* | *pyrG89*, *pyroA4*, *riboB2*, Δ*nkuA*::*argB*; *veA1*; Δ*ST*::*niiA*/*niaD*(p)::*alfR*/*S*::*pyroA*; Δ*yA*::*AN12089*(p)::*pyrG*::*riboB* | this study | *AN12089* test strain |
| TAN17.1* | *pyrG89*, *pyroA4*, *riboB2*, Δ*nkuA*::*argB*; *veA1*; Δ*ST*::*niiA*/*niaD*(p)::*alfR*/*S*::*pyroA*; Δ*yA*::*stcT*(p)::*pyrG*::*riboB* | this study | *stcT* test strain |
| TAAS426.1* | *pyrG89*, *pyroA4*, *riboB2*, Δ*nkuA*::*argB*; *veA1*; Δ*ST*::*niiA*/*niaD*(p)::*alfR*/*S*::*pyroA*; Δ*yA*::*stcU*(p)::*pyrG*::*riboB* | this study | *stcU* test strain |
| TAN18.1* | *pyrG89*, *pyroA4*, *riboB2*, Δ*nkuA*::*argB*; *veA1*; Δ*ST*::*niiA*/*niaD*(p)::*alfR*/*S*::*pyroA*; Δ*yA*::*stcV*(p)::*pyrG*::*riboB* | this study | *stcV* test strain |
| TAAS427.1* | *pyrG89*, *pyroA4*, *riboB2*, Δ*nkuA*::*argB*; *veA1*; Δ*ST*::*niiA*/*niaD*(p)::*alfR*/*S*::*pyroA*; Δ*yA*::*stcW*(p)::*pyrG*::*riboB* | this study | *stcW* test strain |
| TPMW7.2* | *pyrG89*, *pyroA4*, *riboB2*, Δ*nkuA*::*argB*; *veA1*; Δ*ST*::*niiA*/*niaD*(p)::*alfR*/*S*::*pyroA*; Δ*yA*::*riboB* | this study | yellow control strain |
| TPMW8.2* | *pyrG89*, *pyroA4*, *riboB2*, Δ*nkuA*::*argB*; *veA1*; Δ*ST*:: *niiA*/*niaD*(p)::*alfR*/*S*::*pyroA*; Δ*yA*::*riboB*; Δ*wA*::*pyrG* | this study | white control strain |
| TJSF1.1* | *pyrG89*, *pyroA4*, *riboB2*, Δ*nkuA*::*argB*; *veA1*; Δ*ST*::*niiA*/*niaD*(p)::*alfR*/*S*::*pyroA*; Δ*yA*::*riboB*; Δ*wA*::*aflA*/*B*(p)::*carRA*/*B*::*pyrG* | this study | *car* expression strain |
| TJSF2.1* | *pyrG89*, *pyroA4*, *riboB2*, Δ*nkuA*::*argB*; *veA1*; Δ*ST*::*niiA*/*niaD*(p)::*alfR*/*S*::*pyroA*; Δ*yA*::*stcN*(p)::*ggs1*::*riboB* | this study | *ggs1* expression strain |
| TJSF3.1* | *pyrG89*, *pyroA4*, *riboB2*, Δ*nkuA*::*argB*; *veA1*; Δ*ST*::*niiA*/*niaD*(p)::*alfR*/*S*::*pyroA*; Δ*yA*::*stcN*(p)::*ggs1*::*riboB*; Δ*wA*::*aflA*/*B*(p)::*carRA*/*B*::*pyrG* | this study | *ggs1* and *car* expression strain |
|  |  |  |  |

*strains harbor the same secondary metabolite cluster deletions as LO8030

**Table S2. Primer used in this study**

| **Name** | **Sequence** | **Purpose** |
| --- | --- | --- |
| PW_stcW_flank_F | gattacgccaagcttgcatgccggcgcgccgtctgcctctgccagcgagg | aflR/S strain assembly |
| PW_stcW_flank_R | cgtgaataatactaccacagtcttttgcctggagagagcaggcgtgg | aflR/S strain assembly |
| PW_aflS_F2 | caaaagactgtggtagtattattcacg | aflR/S strain assembly |
| PW_aflS_R2 | atggcgactcagccagctggctttttgc | aflR/S strain assembly |
| PW_niiA_F | ccagctggctgagtcgccatgatggcgggcgcggtgattgagc | aflR/S strain assembly |
| PW_niaD_R | ctgatcgctgggggctccatagattactgaagaattcgcagc | aflR/S strain assembly |
| PW_aflR_F2 | atggagcccccagcgatcagcc | aflR/S strain assembly |
| PW_aflR_R2 | ggctgtcgatcatgtggatgctttacttggtgactacctttgttacttgg | aflR/S strain assembly |
| PW_stcA_flank_F | gatgtaagggcgaattctgcacgaaatcaaacgtcgccagcc | aflR/S strain assembly |
| PW_stcA_flank_R | gctgcaaggcgattaagttgggcgcgccttggaattgctgacgctacc | aflR/S strain assembly |
| PW_pyrG-Af-F | atgtcgtccaagtcgcaattg | test strain construction |
| PW_pyrG-Af-riboOL-R | catccataactcagtccgcggcgcgttctgtctgagaggaggcactg | test strain construction |
| PW_stcW_pR | aagtcaattgcgacttggacgacattgcggggggtattgtgtg | test strain construction |
| PW_stcW_pF | tatcagcgaacaacatcaagccgacattgttcttgctgagtac | test strain construction |
| PW_stcV_pR | aagtcaattgcgacttggacgacatattgttcttgctgagtac | test strain construction |
| PW_stcV_pF | tatcagcgaacaacatcaagccgactgcggggggtattgtgtg | test strain construction |
| PW_stcU_pR | aagtcaattgcgacttggacgacatggttgattcctatacctt | test strain construction |
| PW_stcU_pF | tatcagcgaacaacatcaagccgacgtggccgcggctgcgattgc | test strain construction |
| PW_stcR_pR | aagtcaattgcgacttggacgacattgcagaaatattctcaattc | test strain construction |
| PW_stcR_pF | tatcagcgaacaacatcaagccgacgtcgcggcgacctcacaccc | test strain construction |
| PW_stcS_pR | aagtcaattgcgacttggacgacatgggcttgtatcaccagtt | test strain construction |
| PW_stcS_pF | tatcagcgaacaacatcaagccgacggtggagtgaaagtgctg | test strain construction |
| PW_stcT_pR | aagtcaattgcgacttggacgacatggtggagtgaaagtgctg | test strain construction |
| PW_stcT_pF | tatcagcgaacaacatcaagccgacgggcttgtatcaccagtt | test strain construction |
| PW_stcQ_pR | aagtcaattgcgacttggacgacattgtttggttctgtgaggg | test strain construction |
| PW_stcQ_pF | tatcagcgaacaacatcaagccgacggctactgcatgccattc | test strain construction |
| PW_stcP_pR | aagtcaattgcgacttggacgacatggctactgcatgccattc | test strain construction |
| PW_stcP_pF | tatcagcgaacaacatcaagccgactgtttggttctgtgaggg | test strain construction |
| PW_AN12089_pR | aagtcaattgcgacttggacgacatattattgataaacgtgcag | test strain construction |
| PW_AN12089_pF | tatcagcgaacaacatcaagccgacccaatccgactggctctcgg | test strain construction |
| PW_stcO_pR | aagtcaattgcgacttggacgacatcctgatgtaggattagg | test strain construction |
| PW_stcO_pF | tatcagcgaacaacatcaagccgacggcgtagaattctccgaacacg | test strain construction |
| PW_stcN_pR | aagtcaattgcgacttggacgacatagtgacgatggtctgtctg | test strain construction |
| PW_stcN_pF | tatcagcgaacaacatcaagccgacggttttgaagagttccag | test strain construction |
| PW_stcM_pR | aagtcaattgcgacttggacgacatggttttgaagagttccag | test strain construction |
| PW_stcM_pF | tatcagcgaacaacatcaagccgacagtgacgatggtctgtctg | test strain construction |
| PW_stcL_pR | aagtcaattgcgacttggacgacatcctgctagaccatggacc | test strain construction |
| PW_stcL_pF | tatcagcgaacaacatcaagccgaccatctgcctaagcacaaccg | test strain construction |
| PW_stcK_pR | aagtcaattgcgacttggacgacattctctttacaggtcgtc | test strain construction |
| PW_stcK_pF | tatcagcgaacaacatcaagccgactccaataagcgtcttggc | test strain construction |
| PW_stcJ_pR | aagtcaattgcgacttggacgacattccaataagcgtcttggc | test strain construction |
| PW_stcJ_pF | tatcagcgaacaacatcaagccgactctctttacaggtcgtc | test strain construction |
| PW_stcI_pR | aagtcaattgcgacttggacgacattgttgaagcgatctgttg | test strain construction |
| PW_stcI_pF | tatcagcgaacaacatcaagccgacactgaggactcagggggg | test strain construction |
| PW_stcH_pR | aagtcaattgcgacttggacgacatactgaggactcagggggg | test strain construction |
| PW_stcH_pF | tatcagcgaacaacatcaagccgactgttgaagcgatctgttg | test strain construction |
| PW_stcG_pR | aagtcaattgcgacttggacgacatattgagtccgggtgag | test strain construction |
| PW_stcG_pF | tatcagcgaacaacatcaagccgacgccgaataatgttgga | test strain construction |
| PW_stcF_pR | aagtcaattgcgacttggacgacatgccgaataatgttgga | test strain construction |
| PW_stcF_pF | tatcagcgaacaacatcaagccgacattgagtccgggtgag | test strain construction |
| PW_aflS_pR | aagtcaattgcgacttggacgacattactaaaaagtctgtac | test strain construction |
| PW_aflS_pF | tatcagcgaacaacatcaagccgacgatatttgcatatgatac | test strain construction |
| PW_aflR_pR | aagtcaattgcgacttggacgacatgatatttgcatatgatac | test strain construction |
| PW_aflR_pF | tatcagcgaacaacatcaagccgactactaaaaagtctgtac | test strain construction |
| PW_stcE_pR | aagtcaattgcgacttggacgacatggcggcagtacagaactg | test strain construction |
| PW_stcE_pF | tatcagcgaacaacatcaagccgacccaacgcgttcgggacccgc | test strain construction |
| PW_stcD_pR | aagtcaattgcgacttggacgacattttgaagtattgtttaacg | test strain construction |
| PW_stcD_pF | tatcagcgaacaacatcaagccgactgtatagcgaaagttgtg | test strain construction |
| PW_stcC_pR | aagtcaattgcgacttggacgacattgtatagcgaaagttgtg | test strain construction |
| PW_stcC_pF | tatcagcgaacaacatcaagccgactttgaagtattgtttaacg | test strain construction |
| PW_stcB_pR | aagtcaattgcgacttggacgacattggactcgcaatcagag | test strain construction |
| PW_stcB_pF | tatcagcgaacaacatcaagccgaccctggagccgtttattgc | test strain construction |
| PW_stcA_pR | aagtcaattgcgacttggacgacatcctggagccgtttattgc | test strain construction |
| PW_stcA_pF | tatcagcgaacaacatcaagccgactggactcgcaatcagag | test strain construction |
| PW_stcA_F | gattcgctgagctccatgg | northern probe |
| PW_stcA_R | gaaccaaatatacatgctcc | northern probe |
| PW_stcB_F | caacgaaatagtctgccagc | northern probe |
| PW_stcB_R | ctatgtcagtgtaatggcgc | northern probe |
| PW_stcC_F | caagagcatccagaacattg | northern probe |
| PW_stcC_R | ggccttcttcgcaaacctcc | northern probe |
| PW_stcD_F | cccgtctctcagcacccggcc | northern probe |
| PW_stcD_R | caaagaggcccagaaaagcc | northern probe |
| PW_stcE_F | gtttctgtaccagaagttcc | northern probe |
| PW_stcE_R | ccagggcacgtctgagccg | northern probe |
| PW_stcF_F | ccgtctggttcaactgggcg | northern probe |
| PW_stcF_R | ccacagcggtttcttgtccc | northern probe |
| PW_stcG_F | gcccctcgatctcacttgc | northern probe |
| PW_stcG_R | gacatcgaacccagcatcc | northern probe |
| PW_stcH_F | gtatgccggtatatcacgtc | northern probe |
| PW_stcH_R | cgtaaccaacagtactcccg | northern probe |
| PW_stcI_F | gattccaagctatcagagcc | northern probe |
| PW_stcI_R | gatttccgaagaagagtgcc | northern probe |
| PW_stcJ_F | ctcaccttggccgcacctcc | northern probe |
| PW_stcJ_R | gatgatagaggccaacggcc | northern probe |
| PW_stcK_F | ctgtcaactttggcagtcgg | northern probe |
| PW_stcK_R | cggccggctctgcgttacc | northern probe |
| PW_stcL_F | ctttgactccattggcgcgc | northern probe |
| PW_stcL_R | ccgaatttccagatacagc | northern probe |
| PW_stcM_F | gagtaacgctggagtggaagc | northern probe |
| PW_stcM_R | cttcagaactcccaacagc | northern probe |
| PW_stcN_F | ccagggattctccaacggcc | northern probe |
| PW_stcN_R | cagtcgccatgggctggccg | northern probe |
| PW_stcO_F | ccttcgtacgcccttctagg | northern probe |
| PW_stcO_R | ctatatagcagacccccaagc | northern probe |
| PW_stcP_F | cctggaccttttccgcacc | northern probe |
| PW_stcP_R | cccatcatagtaatactcc | northern probe |
| PW_stcQ_F | gccttcctatgcggttctgg | northern probe |
| PW_stcQ_R | cttgtccctctcctttctcg | northern probe |
| PW_stcR_F | caacgagtatttgttcgcg | northern probe |
| PW_stcR_R | caatcttgctgcaacattgc | northern probe |
| PW_stcS_F | gaccggccactttggtgcc | northern probe |
| PW_stcS_R | ggacagctgttgtgtaggg | northern probe |
| PW_stcT_F | cggcactctgtacactcg | northern probe |
| PW_stcT_R | ggcggctgaatcggcatttc | northern probe |
| PW_stcU_F | cgtctcgatggaaaagtcgc | northern probe |
| PW_stcU_R | gccaataatctttccactg | northern probe |
| PW_stcV_F | gcagcgagaagtggctcggg | northern probe |
| PW_stcV_R | cttgtacccctgctttggc | northern probe |
| PW_stcW_F | ccttcagcactagtcgagc | northern probe |
| PW_stcW_R | ctcagtatatcgacatcacc | northern probe |
| PW_aflR_F | gcaacgaaggcaggaccacc | northern probe |
| PW_aflR_R | ggcgtggcggaggatgctg | northern probe |
| PW_aflS_F | cgcctgatggtgactgcagg | northern probe |
| PW_aflS_R | gttcgtatcgcacctctagc | northern probe |
| PW_012089_F | gtcgcggcgacctcacaccc | northern probe |
| PW_012089_R | tgcagaaatattctcaattc | northern probe |
| carB_cF | catcagtgcctcctctcagacagaatggtggggtatagagatgatcag | expression plasmid |
| carB_cR | ttgtccccctctgattgcgagtccaatgagcgacattaagaaatctg | expression plasmid |
| stcAB_cF | tggactcgcaatcagag | expression plasmid |
| stcAB_cR | cctggagccgtttattgc | expression plasmid |
| carRA_cF | tattttcgcaataaacggctccaggatgggctgggaatatgcccaag | expression plasmid |
| carRA_cR | gtcatagtaaagtgattcgcgtcatcgatcattgacgggttcggttggg | expression plasmid |
| ggs1_cF | catcagtgcctcctctcagacagaatggtggggtatagagatgatcag | expression plasmid |
| ggs1_cR | ttgtccccctctgattgcgagtccaatgagcgacattaagaaatctg | expression plasmid |
| stcM_cF | tggactcgcaatcagag | expression plasmid |
| stcM_cR | cctggagccgtttattgc | expression plasmid |

**Table S.3 Plasmids used in this study**

| **name** | **Features** | **Reference** | **Purpose** |
| --- | --- | --- | --- |
| pJMP61 | *pyroA* cassette | (Bok et al., 2013) | cloning |
| pPMW1 | *niiA/niaD::aflR/S; pyroA; riboB; pyrG* | this study | *aflR/S* construct |
| pJSF1 | *stcA/B::carRA/B; pyrG* | this study | *car* expression |
| pJSF2 | *stcM::ggs1; riboB* | this study | *car* expression |
| pANstcA | *stcA::pyrG; riboB* | this study | *stc* promoter test |
| pANstcB | *stcB::pyrG; riboB* | this study | *stc* promoter test |
| pANstcC | *stcC::pyrG; riboB* | this study | *stc* promoter test |
| pANstcD | *stcD::pyrG; riboB* | this study | *stc* promoter test |
| pANaflR | *aflR::pyrG; riboB* | this study | *stc* promoter test |
| pANaflS | *aflS::pyrG; riboB* | this study | *stc* promoter test |
| pANstcE | *stcE::pyrG; riboB* | this study | *stc* promoter test |
| pANstcF | *stcF::pyrG; riboB* | this study | *stc* promoter test |
| pANstcG | *stcG::pyrG; riboB* | this study | *stc* promoter test |
| pANstcH | *stcH::pyrG; riboB* | this study | *stc* promoter test |
| pANstcI | *stcI::pyrG; riboB* | this study | *stc* promoter test |
| pANstcJ | *stcJ::pyrG; riboB* | this study | *stc* promoter test |
| pANstcK | *stcK::pyrG; riboB* | this study | *stc* promoter test |
| pANstcL | *stcL::pyrG; riboB* | this study | *stc* promoter test |
| pANstcM | *stcM::pyrG; riboB* | this study | *stc* promoter test |
| pANstcN | *stcN::pyrG; riboB* | this study | *stc* promoter test |
| pANstcO | *stcO::pyrG; riboB* | this study | *stc* promoter test |
| pANstcP | *stcP::pyrG; riboB* | this study | *stc* promoter test |
| pANstcQ | *stcQ::pyrG; riboB* | this study | *stc* promoter test |
| pANstcR | *stcR::pyrG; riboB* | this study | *stc* promoter test |
| pANstcS | *stcS::pyrG; riboB* | this study | *stc* promoter test |
| pANstcT | *stcT::pyrG; riboB* | this study | *stc* promoter test |
| pANstcU | *stcU::pyrG; riboB* | this study | *stc* promoter test |
| pANstcV | *stcV::pyrG; riboB* | this study | *stc* promoter test |
| pANstcW | *stcW::pyrG; riboB* | this study | *stc* promoter test |
| pAN12089 | *AN12089::pyrG; riboB* | this study | *stc* promoter test |
