# Supplementary material for: CoIN: co-inducible nitrate expression system for secondary metabolites in Aspergillus nidulans
Source: Fungal Biol Biotechnol. 2018 Mar 13;5:6. doi: 10.1186/s40694-018-0049-2 (PMC5851313; doi:10.1186/s40694-018-0049-2)
Supplement: Supplementary file 2 — Additional file 2. Additional figures. [file 40694_2018_49_MOESM2_ESM.docx]

Supplementary Figures


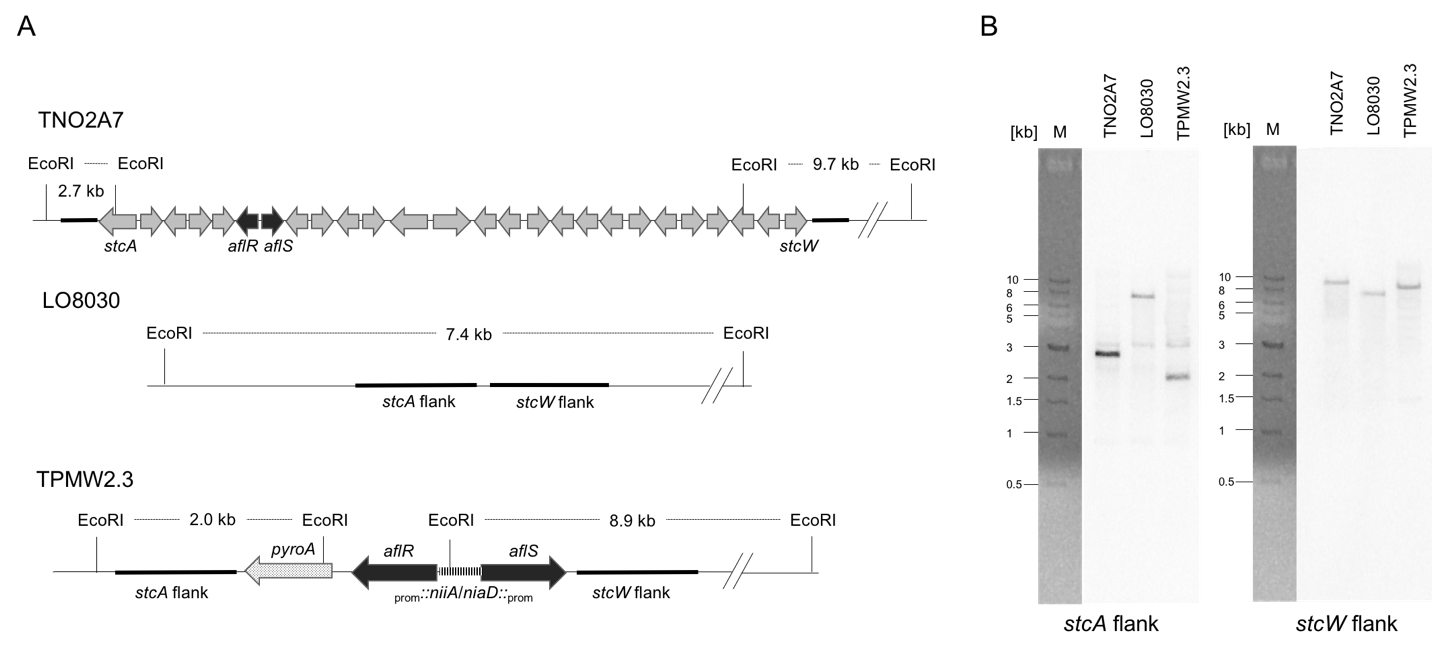


**Figure S1 Integration strategy and Southern analysis of TPMW2.3 harboring the nitrate inducible *aflR*/*S* genes at the native sterigmatocystin cluster locus in LO8030**

1. Integration strategy of the *niaD/niiA*-driven *aflR/S* construct at the sterigmatocystin cluster locus in LO8030. *Eco*RI restriction sites are indicated in all strains and flanks used for hybridization are highlighted in bold.
2. Southern blot analysis of indicated strains. Restriction digest was carried out with *Eco*RI and blots were hybridized with indicated flanks shown in panel A.



**
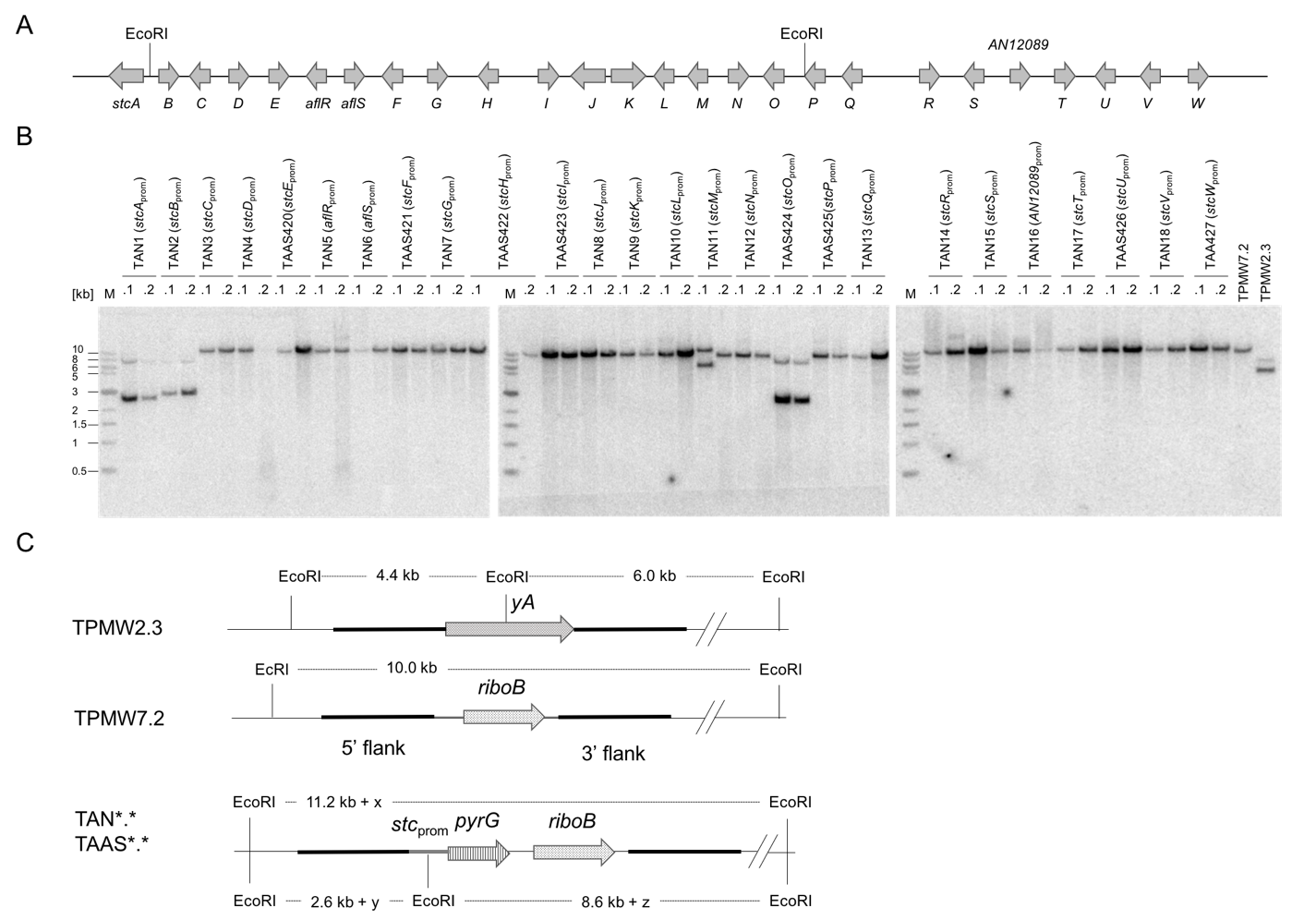
Figure S2 Integration strategy and Southern analysis of *stc* promoter test strains driving *pyrG* intograted at the *yA* locus in TPMW2.3**

1. Schematic overview of *Eco*RI restriction sites within the sterigmatocystin gene cluster in *A. nidulans* TNO27A.
2. Southern analysis of strains harboring each of the *stc* promoters in front of *pyrG*, the recipient strain TPMW2.3. and the control strain TPMW7.2. Restrictions were carried out with *Eco*RI and blots were hybridized with indicated flanks from panel C.
3. Integration strategy of the *stc*-promoter-driven *pyrG* construct at the sterigmatocystin cluster locus in TPMW2.3. EcoRI restriction sites are indicated in all strains and flanks used for hybridization are highlighted in bold. Strains TAN1, TAN2, and TAAS424 harbor an additional *Eco*RI restriction site causing hybridization of two fragments in panel B. Strain TAN11.1 shows an incorrect integration pattern in panel B.


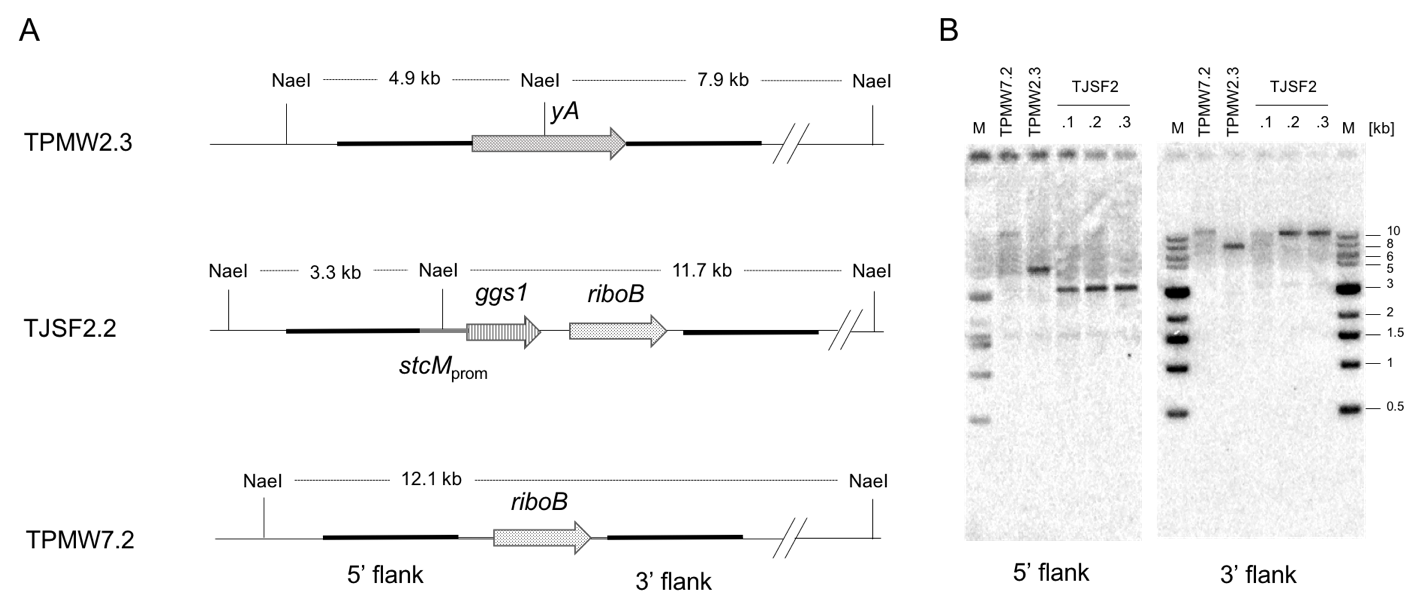


**Figure S3 Integration strategy and Southern analysis of the *A. nidulans* strain TJSF2 harboring the *stcM*-promoter-driven *ggs1* gene from *F. fujikuroi* at the *yA* locus in TPMW2.3**

1. Integration strategy of the *stcM*-promoter-driven *ggs1* gene construct and the control construct at the *yA* cluster locus in TPMW2.3, respectively. *Nae*I restriction sites are indicated in all strains and flanks used for hybridization are highlighted in bold.
2. Southern blot analysis of indicated strains. Restriction digests were carried out with *Nae*I and blots were hybridized with indicated flanks shown in panel A.


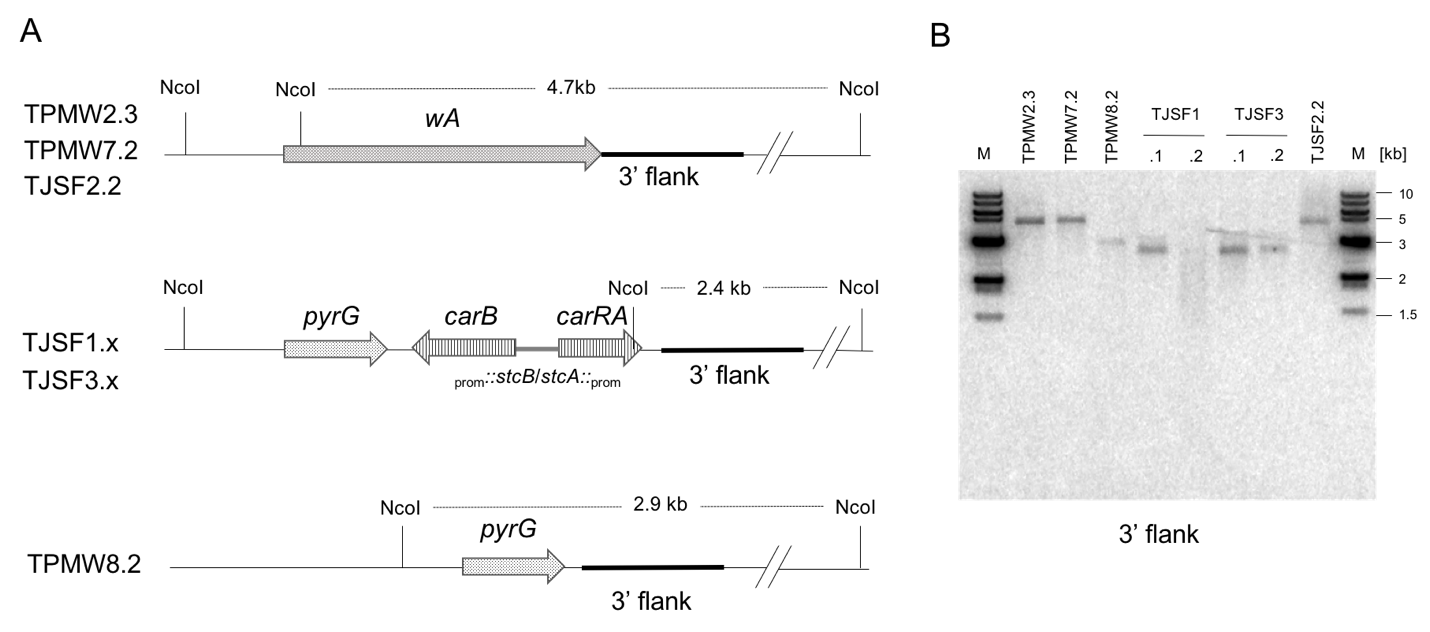


**Figure S4 Integration strategy and Southern analysis of the *A. nidulans* strain TJSF1 and TJSF3 harboring the *stcA*/*B*-promoter-driven *carRA* and *carB* genes from *F. fujikuroi* at the *wA* locus in TPMW7.2 and TJSF2.2**

1. Integration strategy of the *stcA*/*B*-promoter-driven *carRA* and *carB* gene construct and the control construct at the *wA* cluster locus in TPMW7.2 and TJSF2.2, respectively. *Nco*I restriction sites are indicated in all strains and the flank used for hybridization is highlighted in bold.
2. Southern blot analysis of indicated strains. Restriction digest was carried out with *Nco*I and blots were hybridized with the indicated flank shown in panel A.
